# Polyphenol-Modified Starches and Their Applications in the Food Industry: Recent Updates and Future Directions

**DOI:** 10.3390/foods11213384

**Published:** 2022-10-27

**Authors:** Tai Van Ngo, Sandra Kusumawardani, Kannika Kunyanee, Naphatrapi Luangsakul

**Affiliations:** School of Food Industry, King Mongkut’s Institute of Technology Ladkrabang, Bangkok 10520, Thailand

**Keywords:** starch, polyphenol, digestibility, modification method, physicochemical properties

## Abstract

Health problems associated with excess calories, such as diabetes and obesity, have become serious public issues worldwide. Innovative methods are needed to reduce food caloric impact without negatively affecting sensory properties. The interaction between starch and phenolic compounds has presented a positive impact on health and has been applied to various aspects of food. In particular, an interaction between polyphenols and starch is widely found in food systems and may endow foods with several unique properties and functional effects. This review summarizes knowledge of the interaction between polyphenols and starch accumulated over the past decade. It discusses changes in the physicochemical properties, in vitro digestibility, prebiotic properties, and antioxidant activity of the starch–polyphenol complex. It also reviews innovative methods of obtaining the complexes and their applications in the food industry. For a brief description, phenolic compounds interact with starch through covalent or non-covalent bonds. The smoothness of starch granules disappears after complexation, while the crystalline structure either remains unchanged or forms a new structure and/or V-type complex. Polyphenols influence starch swelling power, solubility, pasting, and thermal properties; however, research remains limited regarding their effects on oil absorption and freeze–thaw stability. The interaction between starch and polyphenolic compounds could promote health and nutritional value by reducing starch digestion rate and enhancing bioavailability; as such, this review might provide a theoretical basis for the development of novel functional foods for the prevention and control of hyperglycemia. Further establishing a comprehensive understanding of starch–polyphenol complexes could improve their application in the food industry.

## 1. Introduction

Over the past century, consumer perceptions of the link between food and health have been changing. Modern consumers need not only high-quality, delicious products but also ones that can provide many health benefits. Bioactive food compounds are one of four potential sectors of food innovation in the era of the COVID-19 crisis and the influence of changing consumer behaviors and the agri-food industry [1]. Fruits and vegetables are valuable sources of nutrients and natural bioactive compounds that have antioxidant properties and have been linked to the prevention of aging and related disorders. Furthermore, the COVID-19 pandemic has underscored the effectiveness of polyphenols in maintaining and improving the immune system [2,3]. Accordingly, the health benefits associated with the consumption of phenolic-rich compounds have attracted increasing interest [4,5], as they could support the treatment of many diseases, including cardiovascular diseases, cancer, diabetes, infections, aging, and asthma [6,7,8].

Starchy food products are often associated with chronic diseases such as obesity, diabetes, blood pressure, and heart disease [9]. Consequently, researchers and food companies have directed attention to functional starch-based foods that can support the treatment of chronic diseases. Recent studies have shown that polyphenols can bind to starch or starch-digesting enzymes and reduce starch digestibility in vitro and in vivo [10,11,12,13]. The health benefits and interaction of starch and polyphenol-rich sources have also been investigated in recent years, for example, rice starch complexed with gallic acid or ferulic acid or quercetin, and potato starch which interacts with proanthocyanidins [14], or the combination of maize starch and caffeic acid [15]. The endogenous polyphenols in some nuts are also effective in controlling the blood glycemic index, as shown in the study of Liu et al. [16]. By combining diverse processes and technology, polyphenol-modified starch or flour containing endogenous polyphenols might be processed and converted into functional components to generate these new and promising healthy foods. Such functional ingredients will be beneficial in meeting consumers’ future nutritional and health needs.

Modulating starch with phenolic compounds also changes the functional properties of starch, including its physicochemical properties, pasting properties, thermal properties, and digestibility [16,17]. In particular, changes in the starch structure, granule surface smoothness, relative crystallinity, and short-range molecular order are crucial in reducing digestibility. Changes in organoleptic properties have also been identified when these starches were applied to the food system [18,19]. However, while the physicochemical properties of starch modified with polyphenols have been reviewed in several reports, existing work has mainly focused on the mechanisms by which polyphenols control glycemic index; it remains unclear how starch properties change in relation to the type of starch and phenolic source.

This review provides ten-year recent research information on the changes in physicochemical properties, functional properties, and health benefits associated with diverse types of starch–polyphenol complexes, along with information on the innovative advanced methods being used to produce them. Digital searches of the literature were conducted utilizing a variety of databases, including Google Scholar and PubMed, two popular online research databases, and websites of selected journal publishers such as MDPI, ScienceDirect, and Springer. Some of the common search terms were “starch and polyphenol interaction”, “starch and polyphenol complex,” and “starch and phenolics compounds”. The collecting and summarizing process followed Preferred Reporting Items for Systematic Reviews and Meta-Analyses (PRISMA) principles [20]. A total of 130 published research articles were used to prepare this manuscript. In addition to the abovementioned topics, the applicability of polyphenol-modified starches in the food system was also analyzed, and future research directions are suggested. This review might provide a theoretical foundation and technological guide for the development and production of more novelty starch-based foods in the future.

## 2. General Information about Starch and Polyphenol Composition, Structure, and Properties and the Interaction between Starch and Phenolic Compounds

Starch is a polysaccharide made up of 1,4-linked glucose monomers and hence has a chemical formula of (C_6_H_10_O_5_)_n_. The most basic type of starch is the linear polymer amylose, while amylopectin is its branched counterpart [21]. Starch is stored in the form of chloroplast granules and in storage organs such as cassava roots, potato tubers, and corn, wheat, and rice grains. The botanical origins of these different organs are reflected in the differences in the shape, size, and size distribution of starch granules, their association as individual molecules (simple) or granule clusters (compound), and their composition (α-glucan, lipid, moisture, protein, and mineral content) [22]. In addition to starch, polyphenols can be found in many plants; these compounds contribute to many plant bioactivities as antioxidants and can prevent some health diseases [23,24]. Most polyphenols contain phenolic hydroxyl groups that could interact with starch via non-covalent bonds such as hydrogen bonds and electrostatic and ionic interactions [25,26]. At the molecular level, polyphenol–starch interactions can produce two types of complexes: V-type inclusion complexes with hydrophobic contacts or non-inclusion complexes with weaker binding forces [27]. Recent research has suggested that in the latter type, unique non-covalent connections between polyphenols (mostly anthocyanins, flavonoids, phenolic acids, and tannins) and carbohydrate polymers can be formed via hydrogen bonds and hydrophobic interactions [26]. In addition to their innate activities, polyphenols can affect the nutritional, physicochemical, and digestible qualities of starch, and hence their interactions can be advantageous in preventing type 2 diabetes [28]. For example, polyphenols have been demonstrated to inhibit or slow the action of amylolytic enzymes in the mouth and intestines [29,30]. They can also reduce the activity of pancreatic amylase and glucosidase, thereby inhibiting the breakdown of starches and lowering postprandial blood sugar. Furthermore, in lowering blood glucose, polyphenols exhibit few or no adverse side effects [31]. However, the most recent studies have conducted only in vitro analysis, and more in vivo studies should be considered. In addition, there remains room for optimization of the starch–polyphenol interaction and its functional effects. For one, the types and concentrations of polyphenols determine the inhibitory mechanism [32]. Additionally, the binding interactions between phenolics and free enzymes are responsible for the early stage of the hydrolysis period; the stability and slow-release properties of the starch–polyphenol inclusion complex can be improved to promote increased bioavailability [27,33].

## 3. Properties of Starch–Polyphenol Complexes

**Figure 1:**
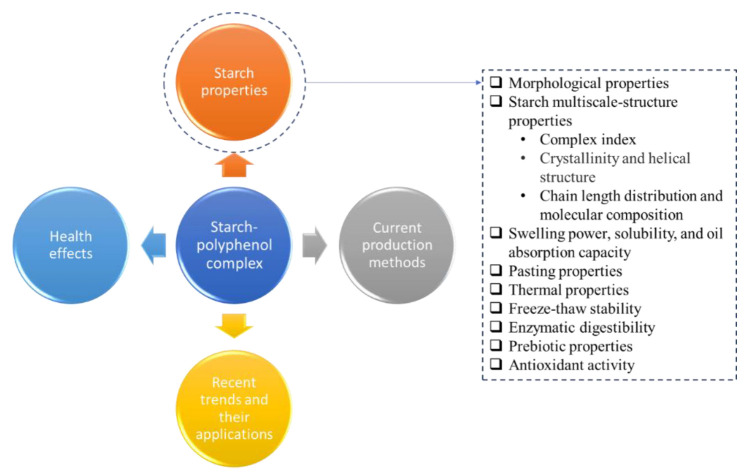
Graphical outline of the information contained in this review.

The modification of starch is usually used to improve functional properties, for example, modifying some physical and chemical properties to facilitate further processing or to meet the needs of current consumers. Recent studies have shown that although starch modification with polyphenols aims to reduce the rate of starch digestion and improve the functional effects of phenolic compounds, this structural modification also inevitably alters starch properties. The most highlighted use of phenolic compounds in modifying starch is to modulate rapidly digestible starch (RDS) into slowly digestible starch (SDS) and resistant starch (RS) while also taking advantage of the functional biological properties of phenolics to improve the immune system. Other properties of starch that could be altered include the aggregate structure, swelling and solubility, gelatinization, crystallization, and pasting, which affect further processing. The properties reported to be altered in starch–polyphenol complexes are listed in Figure 1.

### 3.1. Morphological Properties

As a semi-crystalline biological polymer, starch displays birefringence under polarized light. Starch granules are ovoid, spherical, or irregular in shape and range in size from 1 to 175 µm, depending on the plant [21]. To observe changes in the surface microstructure of starch complexes, scanning electron microscopy (SEM) is commonly applied; field emission scanning electron microscopy (FE-SEM), polarized light microscopy (PLM), and confocal laser scanning microscopy (CLSM) is also used (Table 1).

Significant changes can be observed in many cereal starch granules after complexation under different conditions or with endogenous polyphenols, such as in recent research on maize [15], rice [42], buckwheat [34], potato [17], and acorn kernels [16]. For example, the interaction of caffeic acid with maize starch changed the morphology of the native maize starch from a smooth, spherical structure with a few microscopic pores to a rough surface and angular shape. With increasing concentrations of caffeic acid, more pores were observed in the starch granules [15]. In another study, the removal of polyphenols from acorn flour increased the surface smoothness of starch granules [16]. A similar result was also found in synthesizing phenolic acid cassava starch ester: native starch appeared as spheres or semi-spheres with smooth surfaces, which disappeared after esterification. These findings might be evidence that complexation with a phenolic compound significantly affects the starch granules, especially their smoothness. Additionally, in rice, complexation causes the native starch to be destroyed and form agglomerates during gelatinization. However, complexes have also been found to form a looser porous gel matrix, as in studies of rice starch complexed with ferulic acid/gallic acid/quercetin [42] and wheat starch with lotus leaf flavonoids [42,43]. The presence of anthocyanins has been demonstrated to disrupt the structure of starch granules, as evidenced by a study that mixed rice starch with anthocyanins; more anthocyanin-rich samples displayed a structured network with lots of small holes [43]. Interestingly, in the rice starch–polyphenol–water system, polyphenols may have the capability to hold water [38,43,44]. When flavonoid molecules were caused to enter the molecular cavity of Tartary buckwheat starch, assisted by ultrasound, the complex starch granules became rough with many small holes in the surface. In contrast, a sample processed using high hydrostatic pressure (400 MPa, 1 min) maintained the basic ellipsoidal shape of Tartary buckwheat starch [34].

While the above findings are interesting, there is yet only limited research on particle size, nano-level granule changes, and differences in granule changes among starch modified via different processing methods. Thus, more research should be conducted on nano-level morphology under various conditions and on the effects of starch granule properties on the formation of complexes so as to better understand the effects of complexation.

### 3.2. Starch Multiscale-Structure Properties

The structure of starch determines its physicochemical and functional properties. Currently, techniques commonly applied to identify the interaction of phenolic compounds and starch include diffraction scanning calorimetry (DSC), X-ray diffraction (XRD), and Fourier-transform infrared (FT-IR) spectroscopy [45,46]. Additional methods used to elucidate the formation of starch–polyphenol complexes include isothermal titration microcalorimetry (ITC) [47], cross-polarization magic angle spinning carbon-13 nuclear magnetic resonance (^13^C CP/MAS NMR) [38], and high-performance liquid chromatography (HPLC) coupled with size-exclusion chromatography (SEC) [15,48,49].

#### 3.2.1. Complexation Index

Polyphenols interact with starch through covalent or non-covalent bonds depending on the type of starch and phenolic compound in question. The degree of complexation can be evaluated based on the differential absorbance percentages of native and complexed starch after binding with iodine [42]. The complexation index can also be determined based on the percentage of polyphenols available after complexation as compared to the initial amount of phenolic compounds in an extract [50]. Isothermal titration microcalorimetry (ITC) is also an important technique for studying the interactions of various biological molecules, as illustrated by the research of He, Wang, Zhao, Chen, Zhou, Liu, and Hu [47], which showed a positive entropy (ΔS) longan seed polyphenol to interact with regular maize starch via hydrophobic interactions. Molecular dynamics has likewise been applied to normal wheat starch [51] and revealed that polyphenols with higher molecular weights or many hydrogen bond givers (hydrogen bond acceptors) interact better with amylose.

Physically, polyphenols tend to form compounds with the amorphous hydrophobic area of single helices of amylose. This interaction results in an increased RS level or the formation of a new type of enzyme-resistant starch. Conversely, increasing amylose content in maize starch is negatively correlated with the complexation index between starch and caffeic acid [35]. While amylopectin has a high degree of polymerization, a complex structure, and many side chains, its binding to caffeic acid is facilitated by the formation of hydrogen and conjugated π bonds. Polyphenols can also form non-inclusion complexes through the co-gelatinization of a mixture, or inclusion complexes by complexation of polyphenols with ungelatinized starch, as in the case of wheat starch and tannic acid [52]. Thus, the relationship between starch structure and the rate of complex formation with phenolics is difficult to define and assert; it depends on the starch structure, the type of phenolic compound, and the conditions in which complexation occurs.

#### 3.2.2. Crystallinity and Helical Structure

In recent years, X-ray diffraction has commonly been used to determine starch complexes’ crystalline type and crystallinity. The starch crystal structure is classified as A-type, B-type, C-type (a mixture of A-type and B-type), or V-type based on the X-ray diffraction peaks [53]. Recent research has revealed that the crystalline type of starch depends on its properties; for example, high amylose starch or amylose starch can form V-type starch after complexation, while others remain in the original structure or change to other types or have their crystal structure destroyed, as reviewed and described in Table 2.

For instance, maize starch complexed with longan seed polyphenols exhibited three new XRD peaks at 2θ = 7.5°, 12.7°, and 20.1° (indicating V-type crystal) [47], while combining maize starch with caffeic acid had no effect on starch structure type, with the starch retaining an A-type structure [15]. Interestingly, no clear peaks were observed after the complexation of potato starch and grape seed proanthocyanidins, which indicates the typical crystal was destroyed; only two tiny new peaks were observed at approximately 2θ = 34° and 37.4°. These may indicate that a new structure was formed by this complex [17].

Generally, the crystal structure of the complexed starch is mainly influenced by its amylose content and the type of polyphenol it is complexed with [58]. Research by Obiro et al. [59] showed that the short chains of amylose and amylopectin are unable to produce covalent bonds with polyphenol compounds. Rather, amylopectin and phenolics form non-thermostable amorphous complexes due to hydrogen bonding and electrostatic and ionic interactions [35]. The molecular weight of the polyphenol has also been found to influence its interaction with starch during the modification process. For example, polymeric tannins and proanthocyanidins form indigestible complexes with amylose, but lower molecular weight tannins and monovalent catechins are less able to bind amylose [35,48].

The study of short-range molecular structural changes in starch double helices has made extensive use of IR spectroscopy, as crystalline organized and amorphous structures on the starch surface can be detected by the IR absorbance at 1045 and 1022 cm^−1^, respectively. In addition, the short-range organized molecular structure of starch, including the presence of single and double helical structures, has been characterized using ^13^C CP/MAS NMR. This method can reveal how the starch interacts with other ingredients as well as small molecules (polyphenols, lipids, amino acids) during processing. The number of C1 peaks can be utilized to identify crystal type, while the intensity in the C1 region indicates changes in a starch’s double-helical structure. The amorphous nature of amylose is evident in the C2, C3, and C5 areas (70–79 ppm), which are affected by the amylopectin cluster structure and inversely correlated with the vibrational peak’s intensity [60,61]. The vibrational peaks and regions observed in FT-IR spectra can thus support developing a greater understanding of the complexation between starch and phenolic compounds.

#### 3.2.3. Chain Length Distribution and Molecular Composition

The starch molecules that interact with phenolic compounds are amylose and possibly-linear fragments of amylopectin [62]. When starch is complexed with polyphenols, a change in the length distribution of the amylopectin chain is often noticed. In general, the chain length distribution (CLD) of starch–polyphenol complexes is characterized using a size exclusion chromatography (SEC) system coupled with HPLC [41,47,62]. Changes in the molecular chain structure of starch primarily stem from bond breaks that result in shorter chains or glucose ring vibrations that alter the configuration and conformation of chain segments. For example, the CLD of amylopectin, which peaked at the degree of polymerization 100–1000 was not significantly altered after heating and debranching maize starch with longan seed polyphenols, although a pattern reduced amylose concentration was observed. This suggests that longan seed polyphenols might not interact with amylopectin but rather with amylose [47]. Chai et al. [63] studied the distribution of high amylose maize starch molecule size (without debranching starch molecules) after mixing and heating the starch with tea polyphenols. Tea polyphenols could link multiple amylose molecules together, resulting in the amylose having increased molecular size [63]. However, it merits mention that some linked amylose molecules might be of a size that is beyond the separation limitation of columns and thus cannot be detected by SEC. More research and innovation may therefore be needed to fully elucidate the changes in chain length distribution, and molecule weight during and post-complexation.

### 3.3. Swelling Power, Solubility, and Oil Absorption Capacity

The textural, sensory, and stability characteristics of starch-based food products are influenced by the functional characteristics of the starch in question, such as its capacity to swell, dissolve, and absorb oil. Under specific circumstances, starch can bind water molecules by forming hydrogen bonds between adjacent hydroxyl groups. The ability of starch to retain water at particular temperatures is measured as its swelling power [64]. Amylose functions as a diluent; high amylose concentration or the presence of binding molecules may lower the swelling power [65]. In addition, when starch swells, the diffusion and dissociation of the starch granules cause amylose to be leached [66]. These properties allow the connectivity of starch chains and their interactions with guest molecules in the amorphous and crystalline domains of starch granules to be evaluated using swelling power and solubility. Recent research has investigated the effect of complexation conditions on the swelling power and soluble capacity of wheat starch–tannic acid complexes [52], maize starch–caffeic acid complexes [15], complexes of rice starch with ferulic acid/gallic acid/quercetin [42], amylopectin or potato starch complexed with caffeic acid, gallic acid, or ferulic acid [42,67], and debranched cassava starch–ferulic acid complexes. After complexation, cereal starch’s swelling power and solubility generally showed an upward trend. For instance, in line with the increased amount of apparent amylose, amylose leaching also significantly increased during the complexation of wheat starch and tannic acid, independent of the amount of bound tannic acid [52]. Amoako and Awika [48] likewise reported changes in swelling and solubility for normal (23.9% amylose) and waxy (0.36% amylose) maize starches combined with high tannin sorghum extract: the starch swelled, and pores on the granule surface became channels for phenolic compounds to easily migrate into the interior of the granule, where they could interact with starch polymers. However, some opposite trends have been observed regarding the solubility of starch–polyphenol complexes. For instance, ferulic acid and gallic acid significantly increased the water solubility index of starch [67,68], while increasing concentrations of quercetin produced a downward trend in the water solubility index values of rice starch [42]. Several studies have also shown that physically assisting the complexation also affects swelling properties, for example, through high-pressure homogenization [69,70]. In particular, the shearing forces that occur in high-pressure homogenization are capable of breaking the covalent bonds that link starch chains and hence promote the swelling of granules [71,72]. However, research on the oil absorption capacity of starch complexes remains limited.

### 3.4. Pasting Properties

An instrument commonly used to investigate changes in starch pasting properties is the Rapid Visco Analyzer (RVA) [73]. From the starch science perspective, pasting properties are vital factors that directly affect the quality of a processed product. Recent research has shown that the complexing of starch and phenolic compounds alters the pasting properties such as the peak viscosity, peak time, final viscosity, and setback value (Table 2). For instance, combining black sorghum phenolics with corn starch (23.91% amylose) resulted in a peak viscosity higher than that of native corn starch [74]. This could be due to the additional polyphenols in the solution, which compete with starch for water, or to interactions between the tannins and starch. Additionally, this observation might suggest some interaction between tannins and leached amylose, which could also slow the degradation of starch. Notably, evidence indicates that the polyphenols in white/black sorghum and the proanthocyanidins in tannin sorghum interact with starch via different mechanisms, chiefly due to differences in the molecular weights of the polyphenols involved. For complexes containing caffeic acid, the lower pasting viscosity and temperature observed in comparison to native maize starch may also be attributable to the mild heating temperature used during complexation. However, both peak and final viscosity values significantly decrease with increasing caffeic acid content. This finding suggests that the addition of caffeic acid exerts a greater influence than physical treatment (mild heating), possibly because of the interactions between starch chains and caffeic acid [15]. Green tea polyphenols also significantly reduce the pasting viscosity, trough viscosity, and final viscosity of rice starch in a dose-dependent manner [75]. The authors attributed this to the tendency of tea polyphenols to form hydrogen bonds with starch molecules, which cross-linking could prevent starch granules from expanding during pasting, thereby lowering the pasting attributes. The change of starch molecular occurred, along with the decomposition of molecules that appeared during starch pasting, while starch granules begin to disrupt by amylose exudation at the later stage. However, the recrystallization of the leached starch molecules occurred during cooling.

A recent study introduced the breakdown value, which represents the degree of amylose leaching, and the setback value, which represents amylose and amylopectin retrogradation [76]. Post-complexation setback values of various starch sources are given in Table 3; most decreased, indicating that retrogradation can be retarded by the addition of polyphenols [77]. The promising ability of polyphenols to hinder the retrogradation process is also reflected in the complexes formed by combining maize starch and lotus leaf flavonoids in various ratios [43]. Breakdown values show opposite trends in comparison with setback values, indicating that the complexation process could result in granule stability [77].

### 3.5. Thermal Properties

The thermal characteristics of starch complexes are drastically altered upon the addition of polyphenols, but the effect differs based on the complexation conditions. The influence of changes in the crystalline structure of starch on thermal properties is identified by DSC analysis. Several studies have shown that starch onset temperature (To), peak temperature (Tp), and conclusion temperature (Tc) are all decreased after complexation with polyphenols, in particular, the study of Li et al. [79] on caffeic acid and ferulic acid in complexation with maize amylopectin or potato starch, and that of Lv, Zhang, Li, He, Hao, and Dai [78] on potato starch–tea polyphenol complexes (Table 3). However, combining caffeic acid with maize starch has been reported to increase To, Tp, and Tc [15]. These discordant phenomena could be due to differences in the formation of starch–polyphenol bonds and hence the stability of the new complexes. A study by Zheng, Tian, Kong, Yang, Yin, Xu, Chen, Liu, and Ye [15] also found that gelatinization enthalpy (ΔH) decreases with increasing concentrations of caffeic acid. Specifically, complexes of wheat starch and tannic acid exhibited a lower melting temperature alongside a higher temperature range (T_peak_–T_onset_) and melting enthalpy [52]. Notably, ΔH equates to the melting energy of the starch crystalline region, especially the double-helical structures of amylopectin crystallites [80], as amylopectin is the main component of the crystalline region [81]. Kan, Capuano, Oliviero, and Renzetti [52] explained that a first endothermic transition appears at around 60 °C and is associated with the melting of crystalline amylopectin structures, while a second and third endothermic transition appears at 130–150 °C and is associated with the formation of amylose-tannin complexes. Phenolic compounds may block the stretches of amylopectin and reduce the penetration of amylose [82]. Changes in the crystalline region and redistribution of water in complexes also contribute to the alteration of starches’ thermal behaviors [83].

### 3.6. Freeze–Thaw Stability

Freeze–thaw stability, which may be determined as the amount of water that separates from a gel after freezing and thawing, is a crucial measure of starch structure stability [84]. The retrogradation rate during gel formation is strongly associated with the syneresis rate after freeze–thaw cycles: a higher retrogradation rate usually induces larger amounts of ice, while less water retention ability corresponds to a higher syneresis rate [85]. As mentioned above, the alteration of the physicochemical behaviors of starch could contribute to differences in the freeze–thawing properties. However, our understanding of the structural properties during the freezing and thawing of starch–polyphenol complexes is still limited. One study has shown that the freeze–thaw stability of rice gel is improved by the addition of green tea polyphenols [75] (Table 3), with a reduction in syneresis rate being associated with interference from green tea polyphenols in the starch retrogradation process. In addition, water distribution has a favorable impact on how well starch–polyphenol complexes persist through freeze–thaw cycles [86]. Freeze–thaw treatment can also be used as a novel non-thermal modification technique in its own right as such treatment changes the surface porosity of starch and remodels its crystal topology, thereby increasing the number of functional and binding sites for an added compound to interact with inside the helical cavity of the starch [87]. However, there are few specific and detailed descriptions of freeze–thaw as a pre-treatment for the preparation of starch–polyphenol complexes. Thus, more research should be conducted to continue investigating the changes of starch complexes during freeze–thaw cycles in order to provide more information and knowledge on this treatment’s potential in the enhancement of food quality, especially commercial frozen starch-based foods.

### 3.7. Enzymatic Digestibility

Starch digestibility is one of the principal factors impacting consumer health, especially in the context of diabetes and obesity. It is related to the glycemic index, which is a measure of blood sugar levels after consuming starchy foods. Studies using in vitro methods have shown that starch modified by complexing with phenolic compounds induces a less-rapid increase in glycemic values. Recent studies and updated information indicate that this process can be expressed through two main mechanisms.

Firstly, polyphenols inhibit the amylase enzyme and decrease its activity during starch hydrolysis metabolism [88]. Polyphenol-rich extracts from green tea, black tea [89], oolong tea [90], sorghum [74], Chinese bayberry leaves [37], and longan seeds [47] have all demonstrated competitive inhibition with and the ability to reduce the activity of amylolytic enzymes. However, excessive polyphenol supplementation may oppositely enhance digestibility since heavily-complexed starch has a looser structure and hence offers more opportunities for hydrolysis by digestive enzymes. For instance, when combining starch with caffeic acid, using more than 0.6% acid (that is, 0.8% or 1.0%) results in increased RSD content but decreased SDS and RS contents [15], suggesting that the inhibitory effect of caffeic acid on starch hydrolysis is limited by its concentration. Possibly, the dextrin and disaccharides produced in acid-modified starch could be readily digested by enzymes, and thus the acidic state caused by caffeic acid may accelerate starch hydrolysis [15].

As a second mechanism, phenolic compounds can also effectively inhibit the digestion of starch by forming bonds with the starch molecules; this has been demonstrated in previous studies using tea polyphenols, flavonoids, ferulic acid, quercetin, tea extracts, gallic acid, lotus extracts, proanthocyanidins, tannic acid, p-coumaric acid, and sinapic acid [14,41,42,52,75,88,89,91,92]. Specifically, phenolic compounds form V-type inclusion complexes via hydrophobic interactions with amylose molecules or via hydrogen bonding with amylose or certain amylopectin side chains, thereby inhibiting the enzymatic hydrolysis of the starch molecules [46,52]. In addition, phenolic compounds can bind to starch-digesting enzymes, decreasing enzyme activity and, thus, starch digestibility [32,93]. Both the enhancement and formation of resistant starch and slow digestibility of starch have been reported for starch–phenolic compound complexes [27,52,93]. As these are positive effects of starch complexation, a greater study of the optimization of the complexation process should be considered, as well as whether these effects help ensure the health of consumers. The mechanism of glycemic control by starch–polyphenol complexes also needs to be more comprehensively explored.

### 3.8. Prebiotic Properties

Diet has a significant impact on the composition of the gut microbiota, particularly with regard to host-beneficial bacteria such as *Bifidobacterium* and *Lactobacillus* [94]. Intestinal disorders such as acute gastroenteritis and bowel cancer might be caused by pathogenic microorganisms. Recent studies show that resistant starch, which is found in oligosaccharides and fiber, is not digested in the small intestine; rather, when it reaches the colon, it is fermented by the intestinal microflora, leading to the production of short-chain fatty acids (SCFA) such as acetate, propionate, and butyrate. Therefore, resistant starch is also considered a prebiotic, a non-digestible food ingredient that promotes the growth of healthy bacteria in the gut; both carbohydrate prebiotics and polyphenols are involved in regulating the composition of the gut microbiota [27]. Between its prebiotic properties and the lower glycemic index, resistant starch and its derivatives are suggested as therapeutics for metabolic disorders such as diabetes and obesity [95,96,97]. Starch–polyphenol complexes may achieve the same prebiotic impact as polyphenol-free resistant starch [98] but have the potential added advantage of releasing polyphenols when the resistant starch reaches the large intestine and is used by intestinal microflora. Polyphenol–starch complexes thus have strong prebiotic activity; however, their effectiveness needs to be further validated through in vivo study.

### 3.9. Antioxidant Activity

Polyphenols are naturally occurring, biologically active secondary metabolites of plants present in fruits and vegetables and are receiving increasing attention due to the fact that they possess medicinal effects such as antibacterial, antifungal, and antioxidant properties [99] and also potential technological applications, especially with regard to improving the physicochemical and functional properties of starch. Consuming these compounds at appropriate concentrations could hold promise in the prevention of diseases such as diabetes, obesity, Parkinson’s, Alzheimer’s, and others. Interestingly, the naturally phenolic-rich starch flours such as blue maize, which is rich in ferulic and coumaric acids and anthocyanidins (cyanidin and pelargonidin), support that these benefits can be extractable and applicable. Phenolic compounds from natural sources exhibit significant antioxidant properties through radical scavenging activity; these sources may therefore serve as functional ingredients for the development of prospective nutraceutical foods [100,101], and future food research might benefit from using these materials.

When modified with phenolic compounds, the antioxidant properties of starch are increased, and the in vitro digestibility is decreased, as previously indicated. The addition of phenolic compounds to starch may also delay their release, particularly for those of high molecular weight, into the large intestine (in vivo), where they can scavenge free radicals that are responsible for several disorders [102]. Notably, in the process of starch modification with phenolic compounds, a gradual decrease is observed in antioxidant content as well as in antioxidant activity. This suggests that the decrease in total phenol content may be due to the reaction of phenolic compounds with starch, as shown in the study of Chen, Wang, Xiao, Li, Zhou, and Liu [100]. However, starch–polyphenol inclusion complexes can also enhance polyphenol stability and control polyphenol release, and hence can be regarded as a polyphenol delivery system. Increasing the bioavailability of phenolic compounds in the human body remains difficult; due to the abundance, affordability, biocompatibility, and re-producibility of starch, the starch–polyphenol combination offers a tremendous amount of potential as a polyphenol delivery strategy. In vivo research is still needed to determine this delivery system’s efficiency.

## 4. Current Methods for Producing Starch–Polyphenol Complexes

Food matrix composition, including the presence of polyphenols, affects starch digestibility during food processing. In particular, polyphenol compounds have the potential to delay postprandial blood glucose and reduce starch digestion [32,93]. Non-covalent bonds, such as hydrogen bonds, hydrophobic interactions, or van der Waals forces, can facilitate the binding of polyhydroxy polyphenols to starches and their assembly into V-type inclusion complexes and are responsible for associated alterations in starch structure [103]. Several methods of modifying starch to produce starch–polyphenol complexes have been developed (Table 4). They can be prepared from isolated starch modified with phenolic extract or from natural sources of polyphenols that are also starch-rich. Some preparations utilize non-thermal food processing technology, such as applying high hydrostatic pressure (HHP) to rice starch for complexation with tea polyphenols [38], high-pressure homogenization [57], ultrasound-microwave synergistic processing [41], or microwave irradiation to produce inclusion complexes from lotus seed starch and polyphenols [104]. Other non-thermal treatments, such as a pulsed electric field, pulsed magnetic field, and cold plasma technologies, could also be used. The combination of a non-thermal method with pre-gelatinization is a promising technique to improve complexation [93]; moreover, pre-gelatinization combined with plasma, in particular, could decrease the digestion rate and enhance crystallinity and compactness of starch granules, as demonstrated with starch–quercetin complexes [36].

Chemical and enzymatic methods have also been used to synthesize phenolic starch esters. For instance, Reddy et al. [105] reported that the modification of maize starch–green tea extract complexes by pullulanase debranching and octenyl succinic anhydride produced a maximum recovery yield of 67.81% and increased the stability of bioactive compounds. Oladele et al. [106] produced starch–polyphenol inclusion complexes via pH-based modification, also termed the alkali approach, in which starch and polyphenols are dissolved in KOH or NaOH solutions and subsequently acidified to precipitate the inclusion complex. The starch substantially disintegrates in an alkaline solution, resulting in longer molecules than with partial gelatinization under heating. Chi, Li, Zhang, Chen, Xie, Li, and Bai [103] combined rice starch with varying amounts of gallic acid (4%, 20%, and 50%, *w*/*w*) to modulate the in vitro digestibility, which resulted in increased resistant starch content and decreased the predicted glycemic index. Analysis by Attenuate Total Reflectance Fourier-Transform Infrared Spectroscopy (ATR-FTIR) and Small Angle X-ray Scattering (SAXS) revealed that at lower concentrations, gallic acid might act as a molecular chaperone, allowing starch-ordered structures to reassemble and form, while at higher concentrations, the starch hydrogen bonding networks were broken, and the ordered multi-scale structures of starch gel were diminished. Han, Zhang, Zhang, Huang, Jia, Huang, and Liu [42] likewise generated rice starch–polyphenol complexes using ferulic acid, gallic acid, and quercetin through vortexing and then incubating those mixtures in a water bath at 95 °C for 20 min. All told, additional investigation into and development of processes for the complexation of starch and polyphenols remains needed to characterize alterations to the structure of starch and their effects on starch digestibility.

## 5. Health Effects of Starch–Polyphenol Complexes

Starch digestion begins with salivary amylase enzyme in the mouth when chewing and continues in the stomach, after which the remaining undigested starch, called resistant starch, ferments to form short-chain fatty acids (SCFAs). In the human body, SCFAs are produced by probiotic bacteria in the gut system and might protect against colon cancer [10,11,108]. In general, both internal and external factors affect starch digestibility. Internal factors include the starch granule structure, the ratio of amylose to amylopectin, molecular weight, and the interaction of starch and other components such as protein and intrinsic phenolics. External factors include processing conditions, storage conditions, retrogradation, and gelatinization by heat, which could all reduce the glycemic index (GI) values in food products [109]. Polyphenols might bind and interact with starch, providing many health benefits, and could also inhibit the starch digestion process by modulating amylase enzymes. The complexation of starch and phenolic compounds causes the starch to become more resistant to digestive enzymes and can form new structures or V-type complexes, which confer additional health benefits and especially tend to significantly lower the glycemic index, as mentioned in Section 3.7. Microstructural modification tends to be an effective alternative for delaying starch digestibility in vitro and can be used to develop new, low-GI bread products, thus providing an opportunity to mitigate the global rise in type II diabetes and obesity [18].

Recent studies have shown that black rice anthocyanin extract confers a dose-dependent reduction in starch digestibility, and its use in bread alters starch digestibility not only through enzyme inhibition but also changes in food microstructure. As the prevalence of chronic diseases such as diabetes is increasing and becoming increasingly important worldwide, the link between food and health has been and continues to be of interest. Taking advantage of the interaction between starch and polyphenols could not only help to reduce the incidence of some chronic diseases but also promote polyphenol delivery and improve consumer health through their functional effects [12,13,110,111,112,113,114]. Li, Griffin, Corbin, Neilson, and Ferruzzi [79] investigated the effects of caffeic acid and ferulic acid complexation with maize starch on modulating phenolic bio-accessibility and the glycemic response of starch-based foods in Wistar rats. They observed a positive change in the formation of resistant starch and phenolic entrapment, providing an alternative mechanistic approach for modulating the glycemic properties of starchy foods.

In addition to regulating starch digestion, polyphenols can also regulate blood glucose levels [32]. Regarding testing in vivo on humans, Nyambe-Silavwe et al. [115] showed in a sample of 16 volunteers that plasma glucose was lower after consumption of polyphenol- and fiber-rich food in a dose-dependent manner, determined by reduction in the incremental area under the glucose curve (with decreases of about 27.4–49.0%). Lv, Zhang, Li, He, Hao, and Dai [78] similarly found in a study of eight-week-old male mice that consuming ball-milled potato starch with tea polyphenols slightly reduced the released glucose concentration observed in postprandial glycemic response measurements, while the time of blood glucose peak was longer than without addition of tea polyphenols. In another mouse study using starch and young apple polyphenols, postprandial blood glucose and insulin were lowered by around 10% at peak compared to mice only fed with starch [116]. However, there is still little data regarding the in vivo GI values of starch–phenolic complexes and the products that contain them. In addition, functional products that improve the immune system are of increasing interest to consumers and merit further research.

## 6. Current Trends and Applications

The application of modified starch products is of increasing interest and popularity in the food industry. Modified starch offers functional properties that meet product requirements and provide several health benefits, and as such, it is commonly applied to many commercial products, including bread, pasta, rice noodles, pasta, cookies, dumplings, steam buns, and more. In recent years, starch modified with polyphenols has also been widely studied and developed for use in starch-based products. In general, adding polyphenol-rich extracts/sources results in reduced product digestibility due to the formation of starch complexes via hydrophobic interactions and hydrogen bonds and reduced amylase enzyme activity, as discussed above. For instance, the fortification of bread with anthocyanins from black rice reduced the digestion rate by approximately 12–20%, depending on the amount of dried extract [117,118]. A study by Kan, Oliviero, Verkerk, Fogliano, and Capuano [50] also showed that applying the fortification or co-digestion of bread with berry polyphenols significantly reduced starch digestibility. The same trend was also reported in other studies when various polyphenols were applied, such as tea extract [89], polyphenols from almond hull [119], and anthocyanins from black rice [18]. In addition to nutritional properties, consumer acceptance is a critical factor in determining product quality. Several studies have shown that applying starch–polyphenol complexes not only improved the quality properties but also fostered product acceptance, such as incorporating banana flour into cake [120], carbohydrate cereal bars with polyphenol-rich berries [18], rice cooked with adzuki bean and other anthocyanin-rich sources [113,121,122], and wheat pasta fortified with grape and papaya by-products [19,123].

Notably, fortifying food products with polyphenol sources changes not only dough formation and development but also the physical characteristics of the final product. For instance, caffeic, ferulic, syringic, and gallic acids decrease the mixing time and shearing tolerance of dough [124], while tannin polyphenols could form extensive hydrogen bonds that enhance dough mixing. Meanwhile, anthocyanin-enriched bread has a lower specific volume and greater crumb firmness, supporting a lower extent of gluten development [18]. A recent study by Sui, Zhang, and Zhou [117] likewise supported that supplementing anthocyanin sources into bread leads to a change in dough development. Xu et al. [125] proposed that anthocyanin interaction with SH/SS bonds reduces the resistance and extensibility of dough and weakens the gluten matrix during dough formation.

Starch retrogradation is another vital factor in determining the quality of food. In polyphenol–starch complexes, the interaction between the hydroxyl groups of the polyphenols and the side chains of the starch molecules may prevent starch from retrograding, slowing the recrystallization process and starch aging [15]. The delay of these processes could also be linked to the pasting properties of the product, which were discussed in Section 3.4. For example, proanthocyanin from grape seed delayed the retrogradation of potato starch [126], as did the addition of tea polyphenols to rice starch and starch-based cooked products [127,128].

To summarize, polyphenol–starch-based products not only promise to be beneficial for diabetes with their low digestion rates but also to build immune system function through the presence of phenolic compounds. The interaction between the phenolic compounds and starch changes the microstructure of the starch, as discussed above, and changes in the physicochemical properties, digestibility, and retrogradation were also observed when supplementing with polyphenols. However, the relationship between phenolic compounds and starch, as well as the variation relating to processing and storage in the food system, is still to be elucidated [18]. The influence of food component interactions during the digestion process on polyphenol delivery to the gut microbiota also needs more consideration. Ultimately, it is necessary to study the effect of polyphenol release during product digestion, further develop the production process, and enhance the eating quality of the resultant commercial products [18].

## 7. Future Insights

The use of fruits and vegetables and other natural sources rich in antioxidant compounds such as polyphenols, flavonoids, and their derivatives in preventing and supporting the treatment of several diseases has been widely and deeply studied [4,129]. Polyphenols have also been shown effective in reducing the rate of glucose release during the digestion of flour, starch, or their processed products due to forming new types of starch complexes such as resistant starch and slowly digestible starch products [52,92], as was clearly shown in Section 3.7. Despite several authors proposing mechanisms by which the reduced digestion rate of starches/flours in powder form is reduced, however, there is, as of now, little study of their products. Therefore, more research remains required in this area.

A recent study showed that complexation could also be produced and observed through thermal-assisted treatment; however, some common phenolic compounds, such as anthocyanins, are sensitive to heat processing [130]. On an industrial scale, phenol-modified modification can be obtained through either thermal or non-thermal processing. However, due consideration should be taken when applying starch–phenol complexation in products processed by hot methods such as baking, blanching, pasteurization, and sterilization.

Some starch-rich flours contain natural phenolic compounds, such as blue maize flour, which is rich in ferulic and coumaric acids and anthocyanidins (cyanidin and pelargonidin); these also have potential as functional ingredients for the development of potential nutraceutical foods [101]. In particular, taking advantage of such available polyphenol-rich flours is critically necessary for the development of new low-GI products. Complexation using compounds from natural food sources is both safe and promotes the efficient use of local ingredients. Some agricultural by-products are also excellent sources of polyphenols, and taking advantage of such materials both reduces the burden on the environment and creates more income for farmers to ensure sustainable agricultural production. However, further in vitro studies of starch–polyphenol complexes are needed to characterize the effects of various processing techniques on their functional properties. The current lack of in vitro and in vivo studies on the antioxidant properties of starch–polyphenol complexes also merits addressing. Finally, further studies should also focus on the complete formulation and optimization of starch complex processing and its application in industrial, commercial products.

## 8. Conclusions

In summary, the modification of starch with phenolic compounds changes its physicochemical properties, functional properties, and product characteristics based on the types of phenolic compounds and the starch source. In addition to reducing the digestibility and regulating the glycemic index, complexation also provides some beneficial properties owing to the inclusion of functional phenolics. Utilizing polyphenol sources from by-products to improve the functional properties of starchy products could also promote sustainable agricultural production. However, only limited studies are available concerning prebiotic potential, digestibility when applied in food systems, and the effects of processing techniques on the resulting products. Healthy foods are a focus of future food development, and starch–polyphenol complexes have enormous potential for application within the food industry, but more research is still required to make them commercially feasible. Thus, this research area merits more consideration and promotion.

## Figures and Tables

**Table 1:** Recent studies of the effect of polyphenol–starch complexation on starch morphological properties.

Complex	Analysis Method	Key Findings	Reference
Maize starch + caffeic acid	SEM	Maize starch had a spherical, smooth structure with a few microscopic pores, whereas the complexes had a rough surface and an angular form. More holes were detected when the concentration of caffeic acid was increased.	[15]
Buckwheat starch + flavonoid solution	SEM	Natural buckwheat had an elliptical structure with a smooth surface. During the treatment of the complex starch, small holes appeared, but the original morphology of the starch granules was retained.	[34]
Maize starch + tannin from sorghum	FE-SEM	PA-complexed samples had clearly identifiable granules and granule fragments. However, these granules showed signs of significant enzyme damage from within and a spongy appearance.	[35]
Buckwheat starch + quercetin	SEM	Quercetin bound to starch granules, producing a smoother and more compact grain structure. However, when processed at high temperatures, this structure was easily broken.	[36]
Rice starch + proanthocyanin from Chinese bayberry leaves	SEM, digital camera	Rice grains increased in color intensity with increasing concentrations of Chinese bayberry leaf extract during processing. Grain texture was not significantly affected.	[37]
Rice starch + tea polyphenols	PLM, SEM	Under 400 MPa, starch granules’ morphological and birefringent characteristics tended to deteriorate as tea polyphenol concentration increased, and more structural damage to starch granules occurred.	[38]
Potato starch + proanthocyanins	SEM	The porosity of the starch complex gradually increased with increasing proanthocyanin concentration. When compared with natural potato starch, the microstructure of the grain was smaller and denser.	[14]
Rice, potato, pea starch + polymeric proanthocyanidins	SEM	The addition of polymeric proanthocyanidins changed the surface structure of the starch; specifically, the volume and number of pores were increased, resulting in a more stretched microstructure.	[39]
Lotus seed starch + chlorogenic acid	SEM	The interaction between lotus seed starch and chlorogenic acid made the granule surface rougher.	[40]
Lotus seed starch + green tea polyphenols	SEM, CLSM, laser scattering measurement	Ultrasound-assisted processing caused the complex starch to be broken into smaller pieces. A rough, homogeneous net of microparticles also appeared to cover the surface. CLSM and laser scattering measurements were used to confirm this result.	[41]

SEM, scanning electron microscopy; FE-SEM, field emission scanning electron microscopy; PLM, polarized light microscopy; CLSM, confocal laser scanning microscopy.

**Table 2:** Recent studies of the effects of polyphenol–starch complexation on starch structure.

Starch	Research Methodology	Characterization Method	Key Findings	Crystalline Structure	Reference
Maize starch	Longan seed polyphenols (LSPs): starch (approx. 61% amylose) = 1:5 (*w*/*w*)	FT-IR, WXRD	Maize starch could interact with LSPs through non-covalent interaction. After complexation, new diffraction peaks were seen at 2θ of 7.5°, 12.7°, and 20.1°.	V-type	[47]
	Mixed with caffeic acid (0.2–1.0%, *w*/*w*) in 50 mL of ethanol solution (30%)	XRD, FT-IR	Caffeic acid affects maize starch crystal degree rather than crystal type. The primary force between maize starch and caffeic acid was non-covalent contact.	A-type	[15]
	High-amylose, normal, waxy maize starch mixed with 500 mg caffeic acid	^1^H-NMR, XRD, FT-IR, Iodine binding	The complexation index reduced as the amylose concentration increased. After the incorporation of caffeic acid, the crystallinity was a downward trend, and a V-type crystal formed.	V-type	[46]
	Normal and waxy maize starch complexed with tannins from sorghum	Iodine binding, XRD, FE-SEM	H-bonding helps/stabilizes the semi-crystalline V-complexes formed by high-molecular-weight proanthocyanidins and amylose. Amorphous complexes arise when H-bonding is restricted.	V-type	[35]
Tartary Buckwheat starch	10% flavonoid solution	XRD, FT-IR	Under high hydrostatic pressure treatment, complexation does not change the crystalline type but can increase crystallinity.	A-type	[34]
	Quercetin	XRD, FT-IR	New scattered peaks appeared at 2θ of approximately 12.5°, 13.6°, 19.2°, 21.0°, 23.9°, and 27.3°. Quercetin interacts with starch through non-covalent bonds such as hydrogen bonds during gelatinization. Intermolecular H–H interaction is likely to occur between the two components.	V-type	[36]
Wheat starch	Tannic acid	XRD	Wheat starch’s relative crystallinity is improved by complexation with tannic acid. In all samples containing tannic acid, a new wide peak was identified at a 2θ of 20°.	V-type	[52]
	Tea polyphenols	XRD, FT-IR	Tea polyphenol–wheat starch complexes show broader O-H stretching and C-O-H bending vibrations. Polyphenols from tea likely create hydrogen bonds with wheat starch.	B-type, V-type	[54]
	Epigallocatechin gallate (EGCG)	XRD, FT-IR, Raman spectroscopy	FT-IR and Raman spectroscopy revealed that EGCG and wheat starch form hydrogen bonds. The positions of the diffraction peaks were unaffected by the addition of EGCG; however, their intensity was reduced.	B-type	[55]
Rice starch	Chlorogenic acid	WXDR, FT-IR	Chlorogenic acid enhances the crystallinity of rice starch and mainly interacts with the starch through hydrogen bonds.	V-type	[56]
	Proanthocyanidins from Chinese bayberry leaves	XRD	A new diffraction peak was found at a 2θ of 28°, indicating that Chinese bayberry proanthocyanidins and rice flour may interact in a new way. The crystallinity degree is higher in untreated rice flour.	V-type	[37]
	Anthocyanins from black rice	Iodine binding, XRD, FT-IR, Molecular dynamics, and Molecular docking	Anthocyanins complex with rice starch through a non-covalent connection. A peak observed for rice starch at 17° vanished after anthocyanins were added, indicating a change in the crystalline structure. The broad peak at 3400 cm^−1^ was reduced in intensity, indicating a decrease in the amount of OH groups and bonds among starch molecules.	A-type	[44]
	Tea polyphenols with high-hydrostatic-pressure-gelatinized rice starch	^13^C CP/MAS NMR spectroscopy, XRD	A combination of B + V-type structure was observed in tea polyphenol-treated starch (600 MPa). Disruption of rice starch granules increased with increasing pressure level during pressurization, whereas relative crystallinity decreased.	A-type, B-type, V-type	[38]
Potato starch	Proanthocyanins	FT-IR, XRD	The intermolecular hydrogen-bonding interaction between proanthocyanin and potato starch prevented retrogradation of the starch. After treatment, a V-type diffraction peak replaced the B-type peak.	V-type	[14]
	Grape seed proanthocyanidins (GSP) (0.0–5.0%, based on starch weight)	XRD, FT-IR	There were no distinct peaks in GSP-potato starch complexes, indicating that the long-range crystalline structure had been broken. With the addition of 3.0–5.0% GSP, two additional peaks were discovered at around 2θ of 34° and 37.4°, which indicated the formation of a novel crystal structure.	New crystal structure	[17]
Rice, potato, pea starch	Polymeric proanthocyanidins (PPC, purity ≥ 95%) isolated from grape seeds	FT-IR, XRD	Short- and long-term retrogradation of three starches were both inhibited by PPC. The long-range organized structure of starch was mostly changed through hydrogen bonding.	V-type	[44]
Lotus seed starch	Chlorogenic acid (5%) under microwave gelatinization and hydrotreatment	^13^C CP/MAS NMR spectroscopy, FT-IR	The V-type complex becomes dominant after complete gelatinization (85 °C), cooling, and recrystallization. It acts as a barrier to water and digestive enzymes and inhibits starch enzymatic hydrolysis.	V-type B-type C-type	[40]
	Green tea polyphenols	WXRD	When the pressure was increased to 150 MPa, a V-type crystal structure was formed. Starch fragments took on a “flower-like” form with spherical crystals thickly dispersed throughout their surfaces. LS-GTP complexes exhibited a non-inclusive surface structure with a C-type crystalline structure.	V-type C-type	[57]

**Table 3:** Comparison of pasting properties, thermal properties, and functional properties of starch–polyphenol complexes and corresponding native forms.

Starch	Complexation Condition	Pasting Properties *	Thermal Properties **	Swelling Power (%)	Solubility (g/g)	Freeze–Thaw Stability	Ref.
Corn starch	Native	PV: 4242 BV: 2016 SV: 1944 FV: 4170	-	-	-	-	[74]
	High tannin: 5-20%	PV: 4515.6–4720.8 BV: 1929.6–2162.4 SV: 1636.8–1819.2 FV: 4144.8–4428	-	-	-	-	
Rice starch	Native	PV: 2577 ± 3 BV: 438 ± 7 SV: 619 ± 13 PT: 79.13 ± 0.71	To = 62.23 ± 0.09 Tp = 69.47 ± 0.09 Tc = 78.03 ± 0.17 ΔH = 10.32 ± 0.26	9.55 ± 0.22% at 85 °C	6.22 ± 0.35 at 85 °C	-	[42]
	Ferulic acid: 5–20%	PV: 2383–2605 BV: 1112–1506 SV: −434–−240 PT: 86.70–89.30	To = 61.17 ± 0.05–61.37 ± 0.57 Tp = 68.23 ± 0.09–68.37 ± 0.25 Tc = 76.50 ± 0.14–76.80 ± 0.33 ΔH = 9.62 ± 0.22–10.00 ± 0.05	7.84 ± 0.03– 9.39 ± 0.12% at 85 °C	7.98 ± 0.3 –17.18 ± 0.38 at 85 °C	-	
	Gallic acid: 5–20%	PV: 2338–2584 BV: 1020–1515 SV: −905–−256 PT: 84.58–89.60	To = 48.57 ± 0.05–59.23 ± 0.60 Tp = 55.83 ± 0.05–66.70 ± 0.50 Tc = 63.80 ± 0.57–76.13 ± 0.40 ΔH = 7.54 ± 0.27–11.20 ± 0.25	8.31 ± 0.27–9.91 ± 0.12 at 85 °C	9.27 ± 0.2–21.71 ± 0.46 at 85 °C	-	
	Quercetin: 5–20%	PV: 2779–3301 BV: 464–811 SV: -20–491 PT: 76.98–80.08	To = 62.33 ± 0.12–62.53 ± 0.12 Tp = 69.60 ± 0.33–69.80 ± 0.33 Tc = 77.70 ± 0.16–78.30 ± 0.37 ΔH = 9.19 ± 0.54–10.06 ± 0.03	9.19 ± 0.12–9.33 ± 0.05 at 85 °C	5.56 ± 0.2–6.00 ± 0.05 at 85 °C	-	
Rice starch	Native	PV: 3440 BV: 1923.67 SV: 1449 FV: 2965.33	Tp = 68.59 ± 0.37 ΔH = 12.39 ± 0.32	-	-	~ 35%	[75]
	Green tea polyphenols	PV: 2538–2963 BV: 1484.7–1744.3 SV: 652.00–964.00 FV: 1705.3–2778.8	Tp = 56.28 ± 0.33–64.78 ± 0.10 ΔH = 5.56 ± 0.04–12.39 ± 0.32	-	-	~ 0.2–22%	
Maize starch	Native	PV: 3091 ± 23 BV: 1237 ± 12 SV: 1739 ± 16 FV: 3594 ± 17	To = 59.98 ± 0.95 Tc = 73.10 ± 1.00 Tp = 65.35 ± 0.46 ΔH = 17.12 ± 0.26	~ 17% at 95 °C	~ 14% at 95 °C	~ 60% (5 cycles)	[15]
	Caffeic acid: 0.0–1.0%	PV: 2197–2992 BV: 1184–1452 SV: 773–1722 FV: 1599–3529	To = 60.04 ± 0.42–64.94 ± 0.18 Tc = 73.75 ± 0.57–77.01 ± 0.09 Tp = 65.35 ± 0.40–66.98 ± 0.25 ΔH = 13.46 ± 0.54–16.56 ± 0.32	~ 14–17% at 95 °C	~ 8.0–12.5% at 95 °C	~ 40–55% (5 cycles)	
Potato starch	Native starch	PV: 3496 ± 32.31 BV: 1688 ± 18.99 SV: 449 ± 11.23 FV: 2137 ± 21.23 PT: 63.50 ± 0.16	To = 58.87 ± 0.52 Tp = 62.94 ± 0.46 Tc = 68.32 ± 0.37 ΔH = 11.39 ± 0.37	-	-	-	[14]
	Proanthocyanidins: 2.5–7.5%	PV: 1325–2322 BV: 120–790 SV: 318–415 FV: 1620–1850 PT: 68–69	To = 59.98 ± 0.47–61.40 ± 0.30 Tp = 64.17 ± 0.37–65.99 ± 0.44 Tc = 70.29 ± 0.38–71.80 ± 0.50 ΔH = 9.31 ± 0.09–10.03 ± 0.19	-	-	-	
Rice starch	Native	PV: 2388.67 ± 17.46 BV: 944 ± 3.74 SV: 1114 ± 3.56 FV: 2558.67 ± 22.95	To = 65.61 ± 0.47 Tp = 71.71 ± 0.63 Tc = 80.99 ± 0.55 ΔH = 15.98 ± 0.20	-	-	-	[37]
	Chinese bayberry leaf extract: 0.1–2%	PV: 341.33–427.33 BV: 127.00–223.00 SV: 408.00–424.00 FV: 609.67–639.67	To = 58.49 ± 0.10–61.28 ± 0.87 Tp = 66.00 ± 1.00–67.82 ± 0.28 Tc = 72.61 ± 0.72–77.97 ± 0.51 ΔH = 9.59 ± 0.07–11.41 ± 0.04	-	-	-	
Soluble starch	Native	-	To = 59.95 ± 0.23 Tp = 65.09 ± 0.58 Tc = 73.12 ± 1.10 ΔH = 17.07 ± 1.31	-	-	-	[43]
	Lotus leaf flavonoids crude extract	-	To = 60.81 ± 0.78–61.63 ± 0.45 Tp = 65.43 ± 0.10–67.09 ± 0.58 Tc = 73.27 ± 0.37–73.91 ± 0.33 ΔH = 10.60 ± 0.71–13.23 ± 0.63	-	-	-	
Potato starch	Native	PV: 1600 ± 2.9 PT: 71.6 ± 0.80 BV: 691 ± 6.2 SB: −519 ± 5.1	-	-	-	-	[67]
	Caffeic acid	PV: 951 ± 71 PT: 66.0 ± 1.5 BV: 667 ± 7.0 SB: −617 ± 27	-	-	-	-	
	Gallic acid	PV: 461 ± 13 PT: 68.5 ± 0.057 BV: 242 ± 8.1 SB: −200 ± 9.0	-	-	-	-	
	Ferulic acid	PV: 829 ± 21 PT: 69.6 ± 0.50 BV: 598 ± 16 SB: −561 ± 13	-	-	-	-	
Maize amylopectin	Native	PV: 7070 ± 54 PT: 67.7 ± 0.68 BV: 4780 ± 67 SB: −4430 ± 63	-	-	-	-	
	Caffeic acid	PV: 204 ± 2.6 PT: 50.9 ± 1.1 BV: 145 ± 2.5 SB: −132 ± 2.1	-	-	-	-	
	Gallic acid	PV: 281 ± 2.5 PT: 50.4 ± 0.076 BV: 228 ± 2.5 SB: −212 ± 3.6	-	-	-	-	
	Ferulic acid	PV: 206 ± 3.2 PT: 50.3 ± 0.029 BV: 160 ± 4.6 SB: −147 ± 4.6	-	-	-	-	
Potato starch	Native	-	To = 49.8 ± 0.2 Tp = 84.5 ± 0.4 Tc = 99.6 ± 0.1 ΔH = 992.4 ± 0.3	-	-	-	[78]
	Tea polyphenols	-	To = 60.4 ± 0.1 Tp = 88.0 ± 0.3 Tc = 102.2 ± 0.4 ΔH = 1353.3 ± 0.1	-	-	-	

* The pasting properties of starch were determined using a Rapid Visco Analyzer; PV: pasting viscosity, cP; PT, pasting temperature, °C; BV, breakdown viscosity, cP; SB, setback viscosity, cP; FV, final viscosity, cP. ** To, onset temperature, °C; Tp, peak temperature, °C; Tc, conclusion temperature, °C; ΔH, gelatinization enthalpy, J/g.

**Table 4:** Current methods for producing starch–polyphenol complexes.

Starch	Polyphenol	Type of Modification	Modification Method	References
Rice starch	Tea polyphenols	High hydrostatic pressure treatment	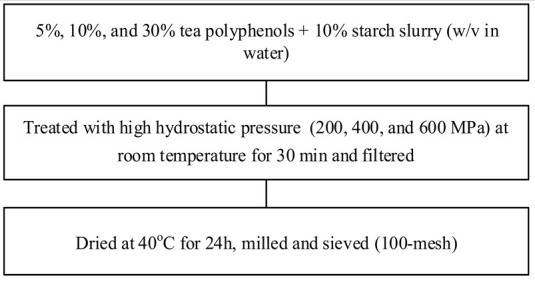	[38]
Lotus seed starch	Green tea polyphenols	High-pressure homogenization	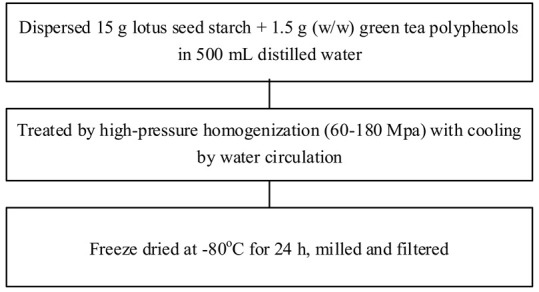	[57]
Lotus seed starch	Green tea polyphenols	Ultrasound-microwave synergistic processing	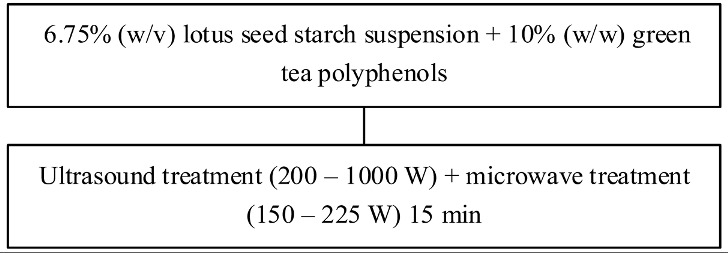	[41]
Lotus seed starch	Chlorogenic acid	Microwave irradiation	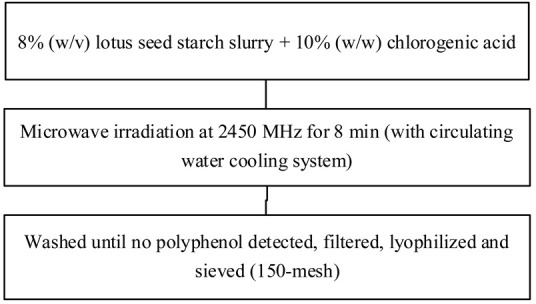	[104]
Tartary buckwheat starch	Tartary buckwheat flavonoids	-	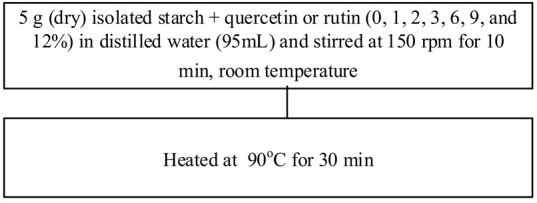	[107]
Tartary buckwheat starch	Quercetin	Plasma Pre-gelatinization	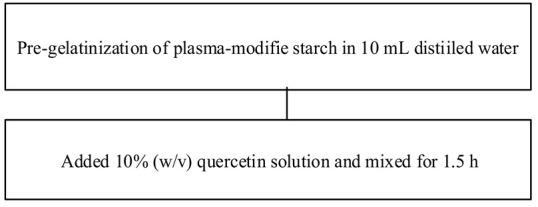	[36]
Rice starch	Ferulic acid, gallic acid, quercetin	-	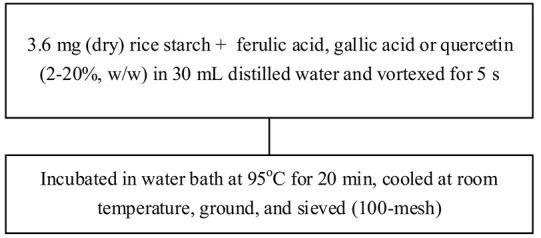	[42]
Rice starch	Anthocyanins	-	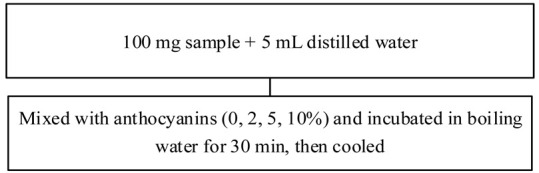	[44]
Maize starch	Grape pomace and sorghum bran	pH-based modification (alkaline condition)	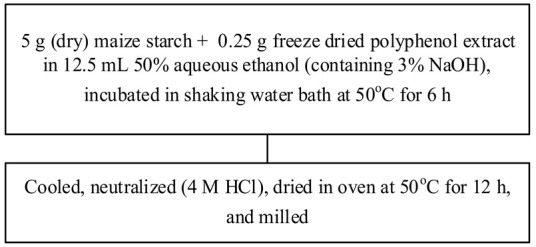	[106]
Maize starch	Green tea extract	Enzymatic modification (Pullulanase debranching or octenylsuccinic anhydride)	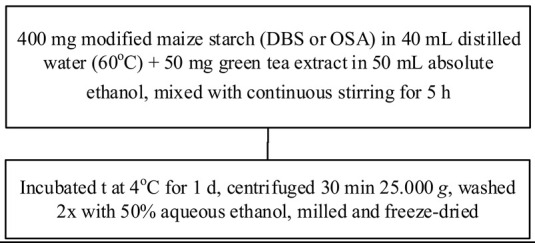	[105]

## Data Availability

Data is contained within the article.
